# Bilateral video-assisted thoracic surgery for esophageal cancer with left inferior pulmonary vein invasion following chemoradiation therapy

**DOI:** 10.1186/s40792-023-01677-w

**Published:** 2023-07-14

**Authors:** Masakazu Fujii, Naoya Okada, Hiroaki Kato, Satoshi Ishihara, Masaru Abe, Takumi Yamabuki, Kentaro Kato, Minoru Takada, Yoshiyasu Ambo, Takeshi Yokoyama, Yoshihiro Kinoshita

**Affiliations:** 1 Department of Surgery, Teine Keijinkai Hospital, 1-12-1-40, Maeda, Teine-Ku, Sapporo, Hokkaido 006-8555 Japan; 2 Department of Anesthesiology, Teine Keijinkai Hospital, Sapporo, Hokkaido Japan

**Keywords:** Bilateral, Esophageal cancer, Lung, Pulmonary vein, Video-assisted thoracic surgery

## Abstract

**Background:**

The surgical strategy for thoracic esophageal cancer that invades the lungs is controversial. In particular, invasion of the pulmonary vein is often regarded unresectable. We successfully applied bilateral video-assisted thoracic surgery (VATS) in esophagectomy for esophageal cancer with left inferior pulmonary vein invasion following induction chemoradiotherapy (CRT), with a favorable response.

**Case presentation:**

A 64-year-old woman was diagnosed with squamous cell carcinoma of the lower third of the esophagus. Computed tomography (CT) revealed that the tumor was suspected to be invading the main trunk of the left lower pulmonary vein and left lower lung. We initiated induction CRT comprising 5-fluorouracil, cisplatin, and concurrent radiotherapy at 50.4 Gy/28Fr. CT revealed shrinkage of the tumor, and the main trunk of the left inferior pulmonary vein was released from the tumor invasion. We considered the tumor to be completely resectable. VATS esophagectomy is usually performed using a right-sided approach. However, the right-sided approach is inappropriate for evaluating tumors around the left inferior pulmonary vein. We started with left-sided VATS to determine tumor resectability and dissected between the esophagus and the main trunk of the left inferior pulmonary vein. We only needed to perform partial resection of the left lower lobe. We then performed a right-sided VATS esophagectomy and lymphadenectomy with partial en bloc resection of the left lower lobe. Following this, we performed hand-assisted laparoscopic proximal gastrectomy and reconstruction using the gastric remnant. The postoperative course was uneventful. The patient was discharged on postoperative day 14. Histopathological examination of the surgical specimen revealed a complete pathological response without any remnant tumor or lymph node metastasis. There were no signs of recurrence or metastasis at the 1-year follow-up.

**Conclusions:**

Curative resection for thoracic esophageal cancer that invades the pulmonary vein could be possible via the bilateral VATS approach following induction CRT with a favorable response.

## Background

In thoracic esophageal squamous cell carcinoma, the primary tumor and its lymph node metastasis with extranodal extension often invade the adjacent critical organs in a narrow mediastinal space. The overall survival of patients with esophageal cancer has improved with surgical techniques and multimodal therapy. However, the treatment of T4 esophageal cancer remains challenging [1]. Video-assisted thoracic surgery (VATS) is the standard esophagectomy approach. Some cases have reported that the bilateral VATS approach is useful for esophageal cancer with para-aortic lymph node metastasis or synchronous pulmonary tumors [2–6]. We report a case of esophageal cancer with left inferior pulmonary vein invasion that was successfully treated with bilateral VATS esophagectomy following induction chemoradiotherapy (CRT).

## Case presentation

**Fig. 1 a:**
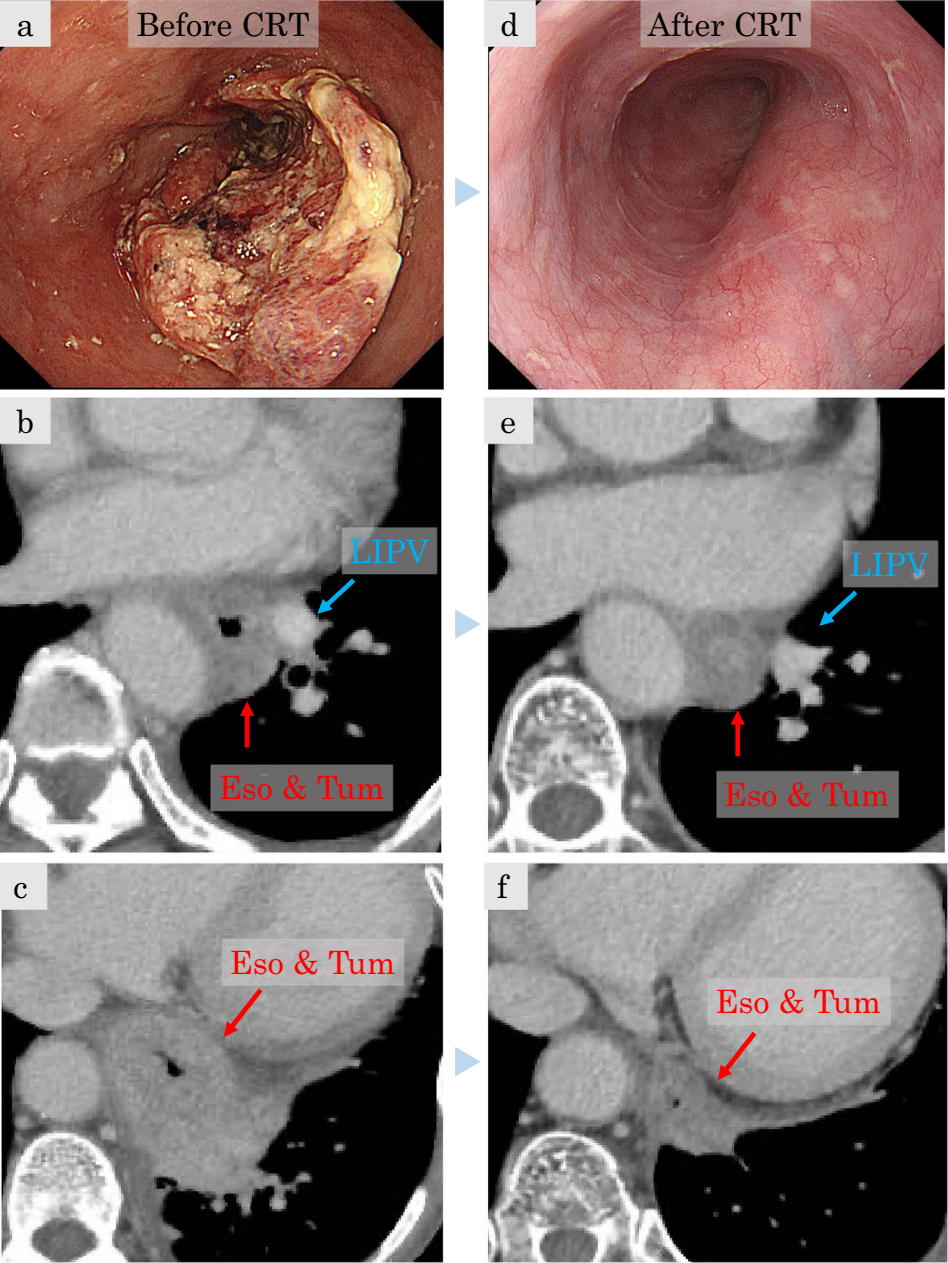
Initial upper endoscopy showing a circumferential type 2 tumor in the lower third of the thoracic esophagus. **b**, **c** Initial contrast-enhanced computed tomography (CT) revealing the tumor extending to the left lower lobe with a suspected invasion of the main trunk of the left inferior pulmonary vein (LIPV) (**b**). Eso & Tum, esophagus and tumor. **d** After induction chemoradiotherapy (CRT), upper endoscopy revealing only the tumor scar with stenosis. **e**, **f** After induction chemoradiotherapy, CT revealing shrinkage of the tumor without any lesion around the main trunk of the LIPV (**e**)

A 64-year-old woman with 3 months of dysphagia was diagnosed with esophageal cancer using endoscopy at a local clinic and was referred to our hospital. The patient had no significant medical history. Endoscopy revealed a 5-cm circumferential lesion in the lower third of the esophagus with stenosis, which was diagnosed as squamous cell carcinoma by tumor biopsy (Fig. 1a). Computed tomography (CT) revealed that the primary tumor was suspected to be invading the main trunk of the left lower pulmonary vein and left lower lung (Fig. 1b, c). Lymph node metastasis was suspected in the left cervical field and mediastinum. The diagnosis of clinical T4b N1 M0 Stage IIIC, as classified by the Union for International Cancer Control tumor-node-metastasis staging system, was established [7].

Induction CRT, comprising two cycles of cisplatin plus 5-fluorouracil for two courses every 3 weeks, was initiated. Cisplatin was administered at a dose of 70 mg/m^2^ via intravenous drip infusion on day 1, and 5-fluorouracil was administered at a dose of 700 mg/m^2^/day via continuous infusion on days 1–5. We also performed concurrent radiotherapy at 50.4 Gy/28Fr. CRT had a significant effect on the tumor. Endoscopy revealed no residual tumor (Fig. 1d). CT revealed a shrunken but remnant tumor with esophageal wall thickness abutting the left lower lobe that was evaluated as partial response according to the Response Evaluation Criteria in Solid Tumors (RECIST) [8]. The main trunk of the left inferior pulmonary vein was released from tumor invasion (Fig. 1e, f). We expected that the tumor would be resectable and considered R0 esophagectomy possible. We planned to perform McKeown esophagectomy with three-field lymphadenectomy and left lung resection that could be a partial or left lower lobectomy. We selected bilateral VATS as the minimally invasive approach. The patient’s preoperative forced expiratory volume (FEV) was 1.4 L.

**Fig. 2:**
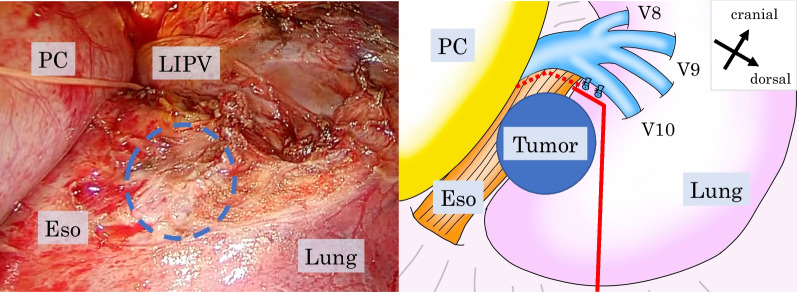
Operative image and schema during the left-sided video-assisted thoracic surgery. The tumor location is drawn with a blue dotted line. We first confirmed that the esophagus could be separated from the main trunk of the left inferior pulmonary vein (dotted red line). The tumor invaded the left lower lobe. Therefore, we ligated the distal pulmonary vein (V10) and performed partial lower lobectomy (bold red line). *Eso* esophagus, *LIPV* left inferior pulmonary vein, *PC* pericardium

**Fig. 3:**
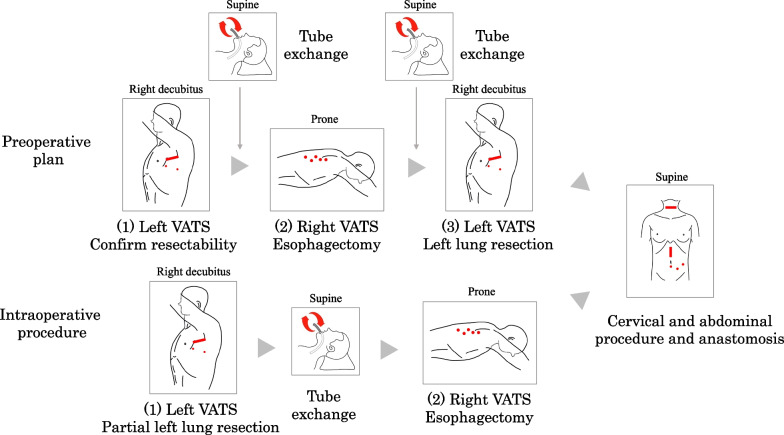
Preoperative strategy and intraoperative procedure. We considered that video-assisted thoracic surgery (VATS) esophagectomy with left lung ventilation could be challenging after left lung resection. Therefore, we preoperatively planned three necessary steps as follows: (1) left-sided VATS only to observe and determine resectability and the range of lung resection, (2) right-sided VATS for esophagectomy, and (3) left-sided VATS for completing lung resection and en bloc esophagectomy. However, the range of lung resection intraoperatively was smaller than we expected, and it would not affect one-lung ventilation during esophagectomy in the right-sided VATS. Our procedure needed only two steps, < 1 > left-sided VATS for partial lower lobe resection and < 2 > right-sided VATS for esophagectomy. We also needed to move the patient to the supine position for tracheal tube exchange

We started with the left-sided VATS in the right decubitus position to determine tumor resectability to check whether the tumor invaded the main trunk of the left inferior pulmonary vein. We dissected the esophagus and the main trunk of the left inferior pulmonary vein. The distal part of the veins (V10) had tumor invasion, requiring only partial en bloc resection of the left lower lobe with the esophagus (Fig. 2). After completing the left thoracic procedure, we extubated the double-lumen tube, reinserted a Nerve Integrity Monitoring (NIM) tube (Medtronic), and placed the bronchial blocker cuff in the supine position. We then performed a right-sided VATS esophagectomy and lymphadenectomy in the prone position. The esophagus was resected and pulled down through the esophageal hiatus, with partial en bloc resection of the left lower lobe via the left thoracic cavity. Following this, proximal gastrectomy was performed via hand-assisted laparoscopic surgery in the supine position, with lymph node dissection around the celiac artery. We also extensively resected the left crus of the diaphragm to secure the tumor margin. The gastric remnant was pulled up through the retrosternal route, and a cervical esophagogastric hand-sewn end-to-end anastomosis was performed (Fig. 3). The operative time was 697 min, and the blood loss was 57 mL.

Although the patient had unilateral recurrent nerve paralysis, the postoperative course was uneventful. The patient was discharged on postoperative day 14. Histopathological examination of the surgical specimen revealed a pathological complete response without any remnant tumor or lymph node metastasis according to the RECIST [8]. She had radiation-induced pericarditis approximately 1 month after surgery. The patient’s condition improved after 1 week of conservative treatment. There were no signs of recurrence or metastasis at the 1-year follow-up.

## Discussion

The treatment strategy for T4 esophageal cancer remains controversial. According to the Japanese national registry of esophageal cancer in 2014, although the number of esophagectomies for esophageal cancer increased, the proportion of esophagectomies for T4 tumors was only 5% [9]. Most T4 cases could be inoperable. R0 resection is essential for a better prognosis even after CRT, with a good response [1]. Especially for unresectable T4b tumors at the first presentation, as in our report, practice guidelines recommend initiating chemotherapy or CRT [10]. When these induction therapies achieve tumor downstaging, patients could be candidates for conversion surgery [11]. Although we selected CRT (cisplatin, 5-fluorouracil, and radiation therapy) as induction therapy in our case, triplet chemotherapy (docetaxel, cisplatin, and 5-fluorouracil) was reported as a potentially more effective therapy. Standardization of induction therapy has not been established, and randomized control trials are ongoing [12].

Surgical outcomes for esophageal cancer with pulmonary invasion were reported in Japan and China before modern multimodal therapy was developed [13, 14]. Few cases of esophagectomy associated with the left inferior pulmonary vein have been reported [15]. Another case report described mediastinoscopy and a left video-assisted thoracoscopic approach for simultaneous esophageal and left lung cancers [16]. However, mediastinoscopy was not feasible in our case because of the highly advanced nature of the tumor. Bilateral VATS is an ideal indication for simultaneous lung tumors in both lobes [17]. This approach has also been reported in three concurrent malignancies in different organs, including the esophagus [2]. In VATS and thoracotomy, the bilateral approach has been reported for esophagectomy with lymph node metastasis around the thoracic aorta [3–6]. To the best of our knowledge, this is the first case report to describe the bilateral VATS approach for thoracic esophageal cancer with left pulmonary invasion.

This bilateral approach required left-sided VATS for left lung surgery in the right decubitus position and right-sided VATS for esophagectomy in the prone position. The proximal portion of the left inferior pulmonary vein was the surgical bottleneck. We would consider the case unresectable if we could not dissect between the tumor and the proximal part of the left inferior pulmonary vein. Thus, we started with the left-sided VATS to determine the resectability of the tumor. Once the tumor was considered resectable, the most extensive lung resection was the left lower lobectomy. In the most extensive lung resection, the patient’s estimated postoperative FEV was 1.4 × 15/19 = 1.1 L. It was satisfactory as more than 0.8 L should be preserved as the minimal postoperative FEV in multiple pulmonary tumor resections [17]. Another problem was that left lung ventilation was necessary for subsequent esophagectomy via VATS. There are no established criteria for lung function, whether the patient could sustain one-lung ventilation. When considering the most extensive lung resection, specifically left lower lobectomy, the anesthesiologist was concerned about left lung single ventilation during subsequent esophagectomy. Therefore, esophagectomy should be performed prior to the removal of the left lower lobe. Our first surgical plan was as follows: (1) left-sided VATS only to determine the resectability and the range of lung resection; (2) right-sided VATS for esophagectomy, leaving the part of esophageal cancer invasion to the left lung; and (3) left-sided VATS for completing lung resection and en bloc esophagectomy via the left-sided VATS (Fig. 3). In this case, we needed only partial left lower lobe resection that would not affect one-lung ventilation; therefore, we could avoid multiple changes in the patient’s position. Changes of the tracheal tube are also important. We always use the NIM in esophagectomy to prevent unintentional recurrent nerve injury and a bronchial blocker cuff to occlude the right bronchus for one-lung ventilation [18]. However, the bronchial blocker cuff is not suitable for lung surgery. We started with a double-lumen tube, reinserted the NIM tube, and placed a bronchial blocker cuff before the esophagectomy. Although the risk of tube replacement is not negligible, tube selection is essential for surgical quality. After the preoperative meeting and simulation, we safely performed the procedure with an anesthesiologist and nurses.

## Conclusion

We successfully performed curative minimally invasive esophagectomy and simultaneous left lung surgery with multimodal therapy for esophageal cancer with invasion of the left inferior pulmonary vein.

## Data Availability

The data supporting the findings of this report are available from the corresponding author upon reasonable request.

## Abbreviations

CRT: Chemoradiotherapy
CT: Computed tomography
FEV: Forced expiratory volume
NIM: Nerve integrity monitoring
VATS: Video-assisted thoracic surgery
